# The Effects of Prenatal Alcohol Exposure on Structural Brain Connectivity and Early Language Skills in a South African Birth Cohort

**DOI:** 10.1162/nol_a_00161

**Published:** 2025-04-02

**Authors:** Mohammad Ghasoub, Chloe Scholten, Bryce Geeraert, Xiangyu Long, Shantanu Joshi, Catherine J. Wedderburn, Annerine Roos, Sivenesi Subramoney, Nadia Hoffman, Katherine Narr, Roger Woods, Heather J. Zar, Dan J. Stein, Kirsten Donald, Catherine Lebel

**Affiliations:** Alberta Children’s Hospital Research Institute, University of Calgary, Calgary, Canada; Hotchkiss Brain Institute, University of Calgary, Calgary, Canada; Department of Radiology, Cumming School of Medicine, University of Calgary, Calgary, Canada; Ahmanson-Lovelace Brain Mapping Center, Department of Neurology, University of California, Los Angeles, Los Angeles, CA, USA; Department of Bioengineering, University of California, Los Angeles, Los Angeles, CA, USA; Division of Developmental Paediatrics, Department of Paediatrics and Child Health, Red Cross Memorial Children’s Hospital, University of Cape Town, Cape Town, South Africa; Neuroscience Institute, University of Cape Town, Cape Town, South Africa; Department of Psychiatry and Mental Health, University of Cape Town, Cape Town, South Africa; Department of Psychiatry and Biobehavioral Sciences, University of California, Los Angeles, Los Angeles, CA, USA; Semel Institute for Neuroscience and Human Behavior, University of California, Los Angeles, Los Angeles, CA, USA; David Geffen School of Medicine, University of California, Los Angeles, Los Angeles, CA, USA; Department of Paediatrics and Child Health, Red Cross War Memorial Children’s Hospital, University of Cape Town, Cape Town, South Africa; South African Medical Research Council (SAMRC), Unit on Child and Adolescent Health, University of Cape Town, Cape Town, South Africa; South African Medical Research Council (SAMRC), Unit on Risk and Resilience in Mental Disorders, University of Cape Town, Cape Town, South Africa

**Keywords:** brain network, connectome, development, language, prenatal alcohol exposure

## Abstract

Prenatal alcohol exposure (PAE) is associated with various neurological, behavioral and cognitive deficits, including reading and language. Previous studies have demonstrated altered white matter in children and adolescents with PAE and associations with reading and language performance in children aged 3 years and older. However, little research has focused on the toddler years, despite this being a critical period for behavioral and neural development. We aimed to determine associations between structural brain connectivity and early language skills in toddlers, in the context of PAE. Eighty-eight toddlers (2–3 yr, 56 males), 23 of whom had PAE, underwent a diffusion MRI scan in Cape Town, South Africa, with language skills assessed using the Expressive and Receptive Communication subtests from the Bayley Scales of Infant and Toddler Development, Third Edition (BSID-III). Diffusion scans were preprocessed to create a structural network of regions associated with language skills using graph theory analysis. Linear regression models were used to examine moderation effects of PAE on structural network properties and language skills. Toddlers with PAE had higher structural connectivity in language networks than unexposed children. PAE moderated the relationship between structural network properties and Expressive Communication scores. None of the effects survived correction for multiple comparisons. Our findings show weak moderation effects of PAE on structural language network properties and language skills. Our study sheds light on the structural connectivity correlates of early language skills in an understudied population during a critical neurodevelopmental period, laying the foundation for future research.

## INTRODUCTION

### Background

Prenatal alcohol exposure (PAE) can negatively impact brain development, causing a range of neurological and physiological deficits (Popova et al., 2023). Alcohol consumption in pregnancy is prevalent worldwide, with approximately 10% of children exposed to alcohol in utero (Popova et al., 2023). Evidence of the teratogenic effects of alcohol on fetal brain development are present throughout neurodevelopment, with alterations to brain structure and function, and their growth over time (Almeida et al., 2020; Lebel et al., 2011; Moore & Xia, 2022). PAE can lead to a wide range of long-term neurobehavioral deficits, and in some cases, a diagnosis of fetal alcohol spectrum disorders (FASD), the neurodevelopmental disorder associated with PAE (Flannigan et al., 2022).

Language deficits are one of the neurobehavioral impairments observed in individuals with PAE (Hendricks et al., 2019; Popova et al., 2016). Several studies have noted phonological awareness and spelling impairments in individuals with PAE (Adnams et al., 2007; Glass et al., 2015). On the other hand, Lindinger et al. (2022) found adolescents with PAE perform poorly on reading comprehension, but not on phonological awareness tasks compared to unexposed adolescents. Several studies found children with PAE have lower expressive and/or receptive language skills compared to unexposed children (Davies et al., 2011; Poth et al., 2023; Proven et al., 2014; Wyper & Rasmussen, 2011), though not all studies show difficulties (Hendricks et al., 2020; Lindinger et al., 2022). Language impairments seem to become more apparent with age, with deficits more commonly reported in older children (Kippin et al., 2021; Proven et al., 2014). Conflicting findings in the literature may also be partly attributed to socioeconomic factors, amount and duration of alcohol exposure, as well as prenatal exposures to other substances that could influence language skills (Flannigan et al., 2022; McLachlan et al., 2020).

Studying the brain’s structural properties can provide a better understanding of the neural basis underlying early language skills in children with and without PAE. Diffusion weighted imaging (DWI) is a neuroimaging technique commonly used to study the brain’s white matter properties due to its sensitivity to white matter microstructure (Beaulieu, 2002). DWI studies consistently show differences in white matter microstructure between typical readers and individuals with reading disabilities, where typical readers tend to have higher fractional anisotropy (FA) and/or lower mean diffusivity (MD) in white matter pathways related to reading and language processing compared to poorer readers (Carter et al., 2009; Lebel et al., 2013; Nikki Arrington et al., 2017; Niogi & McCandliss, 2006; Wang et al., 2021). Recent studies provide evidence for similar relationships between white matter microstructure and early language skills, including receptive and expressive communication, in young children (Feng et al., 2019; Girault et al., 2019; Sket et al., 2019). Travis et al. (2017) found that the FA in the left arcuate fasciculus and superior longitudinal fasciculus were positively associated with phonological awareness in 6-year-old readers and pre-readers. They also found that FA in the right uncinate fasciculus and left superior longitudinal fasciculus were positively associated with receptive and expressive language skills.

In the context of PAE, widespread microstructural brain alterations have been found in pathways associated with language and reading processing in children with PAE (Fan et al., 2016; Gómez et al., 2022; Kar et al., 2021; Lebel et al., 2008; Roos et al., 2021b), but few studies have directly examined associations between brain structure and reading or language performance. One study found that changes over time in MD of the superior fronto-occipital fasciculus were related to changes in reading and receptive vocabulary scores in school-aged children with FASD (Treit et al., 2013). A study by Yu et al. (2022) found significant differences in reading performance and associations with FA of both the superior and inferior longitudinal fasciculi between adolescents with and without PAE. They also found significant differences in the association between lateralization of the inferior longitudinal fasciculus and phonemic decoding performance (Yu et al., 2022). On the other hand, one study found no significant associations between white matter language pathways and reading ability in individuals with FASD (Sowell et al., 2008). Thus, further work is needed to clarify the nature of these associations. Further, there remains a significant gap in the brain–language relationship during the toddler years—when language acquisition is at its peak as most published neuroimaging studies of PAE were conducted in older children. Another significant gap is across diverse socioeconomic and cultural contexts, as most studies have been conducted in high-income countries.

In addition to studying individual white matter pathways, DWI can be utilized to study brain networks associated with language using graph theory, a technique that represents brain regions and the functional and/or structural connectivity between them as a network of nodes connected by edges. This comprehensive map is also known as a connectome (Bullmore & Sporns, 2009; Fornito, 2016). Graph theory has been used to map neural networks from the cellular levels of neurons and synapses to the brain systems levels.

Studies examining structural network connectivity in children reported positive associations between reading performance and global efficiency, a measure of information integration across the network (Lou et al., 2021). Bathelt et al. (2018) found academic performance, measured by reading and math abilities, to be associated with better organization of the white matter connectome, measured by higher global efficiency as well as clustering coefficient, a measure of connection density. Recently, stronger structural network properties were found to be associated with higher phonological processing skills in typically developing preschool-aged children, suggesting that the neural correlates of early reading skills emerge from a young age (Ghasoub et al., 2024a).

Few studies have investigated the structural brain connectomes of children with PAE. One study found no global connectivity differences between neonates with PAE and unexposed infants, though at the regional level, neonates with PAE had lower connectivity in parietal regions and higher connectivity in frontal, occipital, and temporal regions compared to unexposed neonates (Roos et al., 2021a). Another study found overall decreased structural connectivity in the attention, somatomotor, and default-mode networks in children and adolescents with PAE compared to unexposed children and adolescents (Long et al., 2020). Moreover, decreased structural connectivity and network organization in the reading network, measured by global efficiency, nodal degree, and clustering coefficient are associated with poorer phonological awareness skills in preschool-aged children with PAE (Ghasoub et al., 2024b). Nonetheless, most prior studies were conducted on children living with non-birth families with high socioeconomic status. Furthermore, the extent to which these structural connectivity alterations in PAE play a role in early expressive and communication language skills, as well as the presence of these associations in the toddler years, remain to be fully understood.

### The Current Study

This study aimed to investigate the structural network connectivity in language networks, how it is associated with early receptive and expressive language skills in a cohort of South African toddlers (age 2–3 yr), and the extent to which PAE moderates these associations.

## MATERIALS AND METHODS

### Participants

Eighty-eight children aged 2–3 years (2.75 ± 0.14 years, 56 males) from the Drakenstein Child Health Study (DCHS), a population-based longitudinal birth cohort study conducted in the Western Cape region of South Africa (Donald et al., 2018; Stein et al., 2015), were included in this study. A total of 121 children from the DCHS were initially recruited for magnetic resonance imaging (MRI) scans at ages 2–3 years. Thirty-three datasets were excluded from the analysis for poor quality (28 for motion artifacts and 5 for connectomes that were not fully connected), resulting in the final sample size of 88 participants. Twenty-three children had PAE) confirmed via the Alcohol, Smoking, and Substance Involvement Screening Test (ASSIST). None of the children with PAE in our sample had an FASD diagnosis, as assessments for FASD are typically conducted at older ages in South Africa (>6 yr). Prenatal tobacco exposure (PTE) was reported in 14 of the 23 PAE participants and 11 of the 55 controls. Children with confirmed exposures to other drugs (e.g., cannabis, cocaine, opiates, methamphetamine, or barbiturates) were excluded from the study. Infants with congenital malformations, born prematurely, and twins or triplets were also excluded. The Western Cape region is culturally and linguistically heterogeneous. The children spoke primarily Afrikaans or isiXhosa as their native language. Informed parental/guardian consent was obtained from all participants.

### Language Assessments

Child development was assessed objectively by trained assessors using the Bayley Scale of Infant Development, third edition (BSID-III; Bayley, 2012). The BSID-III is validated and standardized using a representative sample of the United States population and has demonstrated its reliability for use in the South African population (Rademeyer & Jacklin, 2013). The Expressive Communication and Receptive Communication subsets of the BSID-III were used to assess early language skills. *Expressive Communication* describes the child’s ability to communicate their thoughts and feelings, which includes gesturing and naming objects. *Receptive Communication* describes the process of receiving and understanding language, which includes the child’s ability to recognize words and objects. Assessments were translated from English (forward and back translation) and conducted in the child’s native language with the assessors blinded to the child’s exposure status. For both Expressive and Receptive Communication, scales scores were used; these have a range of 1–19 and a mean score of 10 ± 3.

### Image Acquisition and Processing

All MRI data was acquired on a 32-channel head coil Siemens Skyra 3T MRI scanner (Siemens, Erlangen, Germany) at Groote Schuur Hospital in Cape Town, South Africa. DWI data acquisition was done using voxel size 1.8 × 1.8 × 2.0 mm, TR = 7,800 ms; TE = 92 ms, slice thickness of 2 mm, with 30 gradient diffusion directions at b = 1,000 s/mm^2^, and one b = 0 s/mm^2^. One full diffusion imaging protocol was acquired in each of the anterior-posterior and posterior-anterior phase encoding directions. Participants were not sedated and were sleeping naturally during the scans. DWI data were preprocessed using ExploreDTI (Leemans et al., 2009) to flip/permute images, and correct for Gibbs ringing, head motion, and eddy current distortions. The diffusion tensor was calculated, and FA values were extracted. Whole brain tractography was performed with seedpoint resolution 2 × 2 × 2 mm^3^, FA threshold = 0.15, fiber length = 50–500 mm, angle threshold = 30°, step size = 1. DWI data of each participant were manually inspected for quality control and to ensure their usability for the following analyses. This step resulted in the exclusion of 28 scans from the initial 121 datasets.

### Connectome Construction and Graph Theory Analysis

The brain was parcellated into 90 regions, excluding the cerebellum, using the Automated Anatomical Labeling atlas (AAL; Tzourio-Mazoyer et al., 2002). AAL gray matter regions were dilated by 3 mm to ensure white matter tracts could reach AAL regions using FSL (Jenkinson et al., 2012). Binarized connectivity matrices of the brain regions of interest were generated using the 90 regions from the AAL and whole brain tractography with ExploreDTI (Leemans et al., 2009), where the parcellated regions represented the nodes of the graph. If the average FA of streamlines between nodes was above 0.15, the edge between these nodes was included in the resultant (binarized) connectome. The connectivity matrices were manually inspected for quality control to ensure that the regions of the network were fully connected, and five scans were found to not have a fully connected network and were thus excluded. This resulted in our final sample size of 88 datasets.

**Figure 1.**
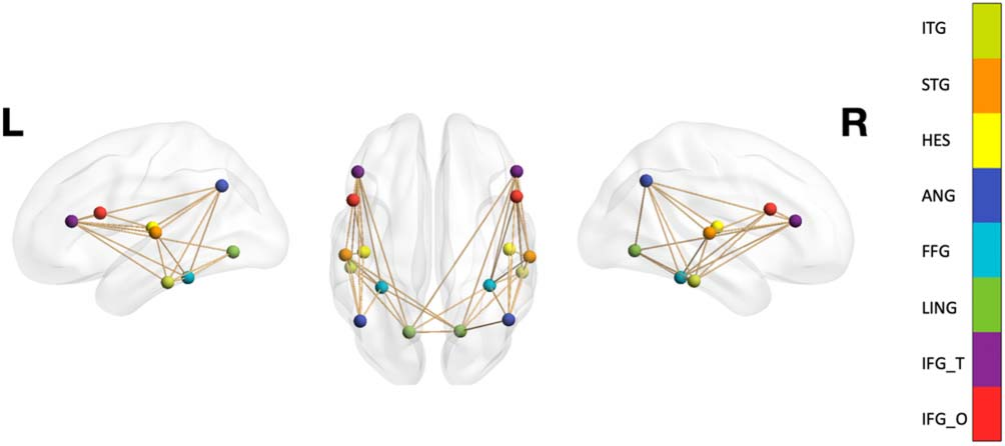
Left, bilateral, and right hemisphere language networks were defined as including the inferior temporal gyrus (light green; ITG), superior temporal gyrus (orange; STG), Heschl’s gyrus (yellow; HES), angular gyrus (navy blue; ANG), fusiform gyrus (sky blue; FFG), lingual gyrus (green; LING), triangular part of the inferior frontal gyrus (purple; IFG_T), and opercular part of the inferior frontal gyrus (red; IFG_O). Figure was generated using BrainNet Viewer (Xia et al., 2013).

Brain regions that have well-established associations with language skills (Hertrich et al., 2020; Martin et al., 2015; Turker et al., 2019) were selected to form a network for analysis: the opercular part of the inferior frontal gyrus, the triangular part of the inferior frontal gyrus, the lingual gyrus, the fusiform gyrus, the angular gyrus, Heschl’s gyrus, the superior temporal gyrus, and the inferior temporal gyrus (Figure 1). Moreover, PAE has been recently reported to moderate the associations between this network and other language skills (Ghasoub et al., 2024b), further supporting their selection for analysis. The connectivity matrix of this network was extracted from the whole brain matrix.

Graph theory measures of network connectivity (clustering coefficient, global efficiency, local efficiency, nodal degree, and betweenness centrality) were calculated at the selected language network level using the Brain Connectivity Toolbox (Rubinov & Sporns, 2010). These measures have been previously related to reading skills in older children (Mao et al., 2021; Vourkas et al., 2011). *Clustering coefficient*, a measure of functional specialization, describes the average density of connections between regions. *Global efficiency* measures the efficiency of information transfer across the whole network, and *local efficiency* measures average information transfer efficiency between local neighboring nodes. *Nodal degree* is the average number of connections (edges) projecting to/from each region. *Betweenness centrality* measures the average node’s role in acting as a bridge between separate clusters across the network. All five metrics were calculated for the bilateral network (16 × 16 matrix), as well as the left and right networks separately (8 × 8 matrices).

### Statistical Analysis

Statistical analysis was conducted using R studio (Version 4.2.1; R Core Team, 2021). *T* tests were used to test group differences in age, Expressive Communication scaled scores, and Receptive Communication scaled scores. Differences in sex and household income distributions between groups were tested using chi-squared tests. Two linear regression models were used to examine PAE’s effects on graph theory metrics and language scores. The first regression model examined differences in graph theory metrics between children with PAE and controls while controlling for age, sex, and household income with the following equation: Graph Theory Metric ∼ Sex + Age + Household Income + PAE. We then used a second model to determine how PAE moderated the association between language skills and graph theory metrics using the following equation: language ∼ Sex + Age + Household Income + PAE + Graph Metric + Graph Metric * PAE. Initially, native language was included as a covariate in the regression models but was removed as it did not have significant effects and did not contribute to the fit of the model. Household income had significant effects and contributed to the model fit, therefore was included as a covariate. Given its prevalence in this dataset, we also ran the same models with PTE included to examine its effects. Separate models were used to test each graph theory metric (clustering coefficient, global efficiency, local efficiency, nodal degree, betweenness centrality) from each network (bilateral, left, right), with each language measure (Expressive Communication, Receptive Communication). Results are reported both uncorrected and corrected for multiple comparisons using false discovery rate (FDR) at *q* < 0.05.

### Supplementary Analysis

In addition to the network selected for the main analysis, we examined the PAE moderation effects on the brain–language associations in an extended network that included the same regions of interest with the addition of the middle temporal gyrus and supramarginal gyrus. The results of this analysis are reported under the supplementary materials, available in the Supporting Information at https://doi.org/10.1162/nol_a_00161.

## RESULTS

### Group Differences in Demographics and Language Performance

Children in the PAE group were younger than the control group by just under 1 month (*p* = 0.02). There were no significant differences in sex or household income distribution between groups. Expressive Communication and Receptive Communication scores did not differ significantly between the PAE and unexposed groups (Table 1). Given that age was significantly different, we re-ran the language models to control for age as a covariate only and then added a group-age interaction. In these models, there were still no significant group differences in Expressive or Receptive Communication scores.

**Table 1.** Participant demographics and language scores

	PAE (*n* = 23)	Control (*n* = 65)	*p* value
Age (months)	32.57	33.52	0.02*
Sex	Male = 13 (56.5%)	Male = 43 (66.2%)	0.57
Household income	<R1,000/month = 8	<R1,000/month = 13	0.29
	R1,000–5,000/month = 13	R1,000–5,000/ month = 41	
	>R5,000/month = 2	>R5,000/month = 11	
Expressive Communication scaled score (*n* = 76)	6.86	7.38	0.45
Receptive Communication scaled score (*n* = 78)	6.71	6.93	0.68

*Note*. *T* tests were used to test group differences in age and Expressive and Receptive Communication scores. Chi-squared tests were conducted to test group differences in sex and household income.

* Indicates statistically significant (*p* > 0.05); 1 South African Rand (R) = ∼0.054 US dollar.

### Group Differences in Graph Theory Metrics

**Figure 2.**
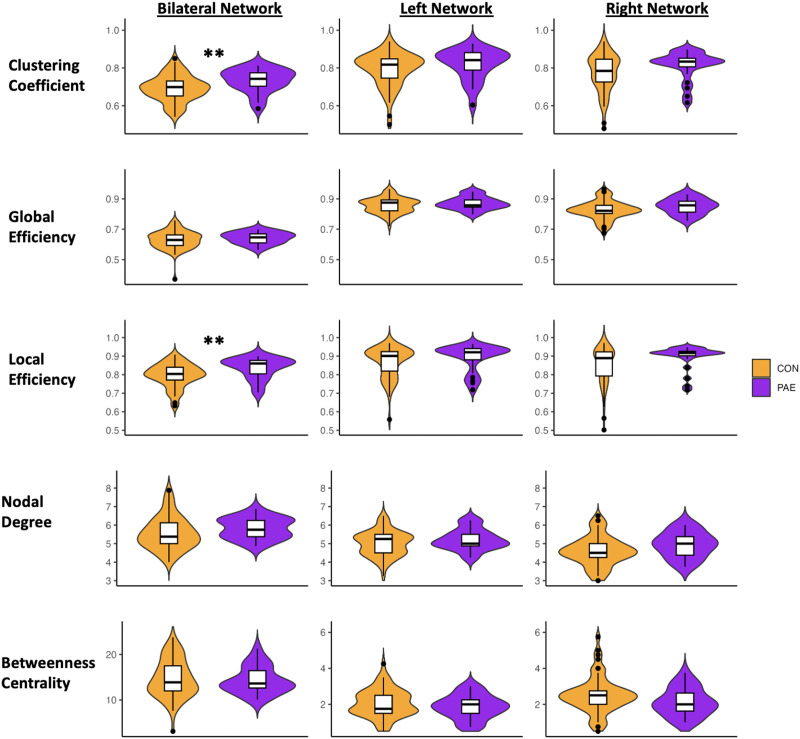
Differences in graph theory metrics between prenatal alcohol exposure (PAE) and control (CON) groups. Clustering coefficient (top row), global efficiency (second row), local efficiency (third row), nodal degree (fourth row), and betweenness centrality (fifth row) in the bilateral network (16 × 16 matrix, first column), left network (8 × 8 matrix, second column), and right network (8 × 8 matrix, third column). The PAE group had higher clustering coefficient (*p* = 0.008) and local efficiency (*p* = 0.008) in the bilateral network compared to controls. ** indicates significant differences (*p* < 0.01); however, these differences did not survive multiple comparison correction (*q* < 0.05).

Children with PAE had higher average local efficiency (beta = 0.04, 95% CI [0.01, 0.07], *t* = 2.71, *p* = 0.008, *q* = 0.060) and clustering coefficient (beta = 0.05, 95% CI [0.01, 0.08], *t* = 2.73, *p* = 0.008, *q* = 0.060) in the bilateral language network compared to unexposed children (Figure 2). These differences did not survive multiple comparison correction.

### Language Assessments

**Figure 3.**
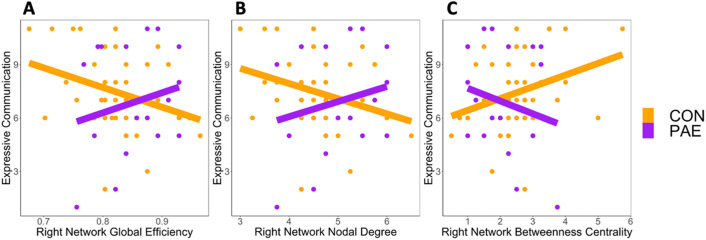
PAE moderated the Expressive Communication–graph theory metric associations for (A) global efficiency, (B) nodal degree, and (C) betweenness centrality in the right hemisphere network.

Regression models for Expressive Communication revealed significant moderation effects of PAE on communication–graph theory metric associations for right hemisphere network global efficiency (beta = 22.70, 95% CI [1.57, 43.83], *t* = 2.14, *p* = 0.036, *q* = 0.205), nodal degree (beta = 1.70, 95% CI [0.13, 3.27], *t* = 2.15, *p* = 0.035, *q* = 0.205), and betweenness centrality (beta = −1.44, 95% CI [−2.83, −0.06], *t* = −2.08, *p* = 0.041, *q* = 0.205; Table 2). In these models, children with PAE had a positive relationship between Expressive Communication and global efficiency and nodal degree and a negative relationship between Expressive Communication and betweenness centrality, while the opposite relationship was found in the unexposed children (Figure 3). None of the significant moderations survived FDR corrections. Additionally, there were main effects of global efficiency (beta = −11.72, 95% CI [−21.82, −1.63], *t*(69) = −2.32, *p* = 0.023, *q* = 0.125), nodal degree (beta = −0.88, 95% CI [−1.64, −0.11], *t*(69) = −2.29, *p* = 0.025, *q* = 0.125), and betweenness centrality (beta = 0.73, 95% CI [0.10, 1.37], *t*(69) = 2.32, *p* = 0.023, *q* = 0.125) on Expressive Communication scores; these did not survive FDR correction. Children with PAE had lower Expressive Communication scores in these models (Table 2). There were no significant sex or household income effects in any of the models. No significant moderation effects were found in the rest of the regression models or Receptive Communication scores (Table 3).

**Table 2.** Associations between graph theory metrics and Expressive Communication scores

Predictors	Bilateral			Left			Right		
	*β*	CI	*p*	*β*	CI	*p*	*β*	CI	*P* (*q*)
Clustering coefficient									
Age	0.30	[−0.03, 0.63]	0.073	0.28	[−0.04, 0.61]	0.089	0.37	[0.03, 0.70]	0.031 (0.105)
PAE status	−9.19	[−22.68, 4.30]	0.179	−4.03	[−15.67, 7.60]	0.492	−4.83	[−16.69, 7.03]	0.419
Clustering coefficient	−0.37	[−9.42, 8.68]	0.935	3.68	[−2.86, 10.23]	0.265	−5.39	[−12.09, 1.32]	0.113
PAE status × Clustering coefficient	12.39	[−6.11, 30.88]	0.186	4.70	[−9.54, 18.93]	0.512	6.06	[−8.61, 20.73]	0.413
Global efficiency									
Age	0.31	[−0.01, 0.64]	0.057	0.27	[−0.06, 0.60]	0.108	0.38	[0.06–0.70]	0.019 (0.105)
PAE status	−14.37	[−33.43, 4.68]	0.137	−13.77	[−36.81, 9.27]	0.237	−19.15	[−37.06, −1.23]	0.037 (0.260)
Global efficiency	−9.02	[−20.39, 2.35]	0.118	3.71	[−8.02, 15.44]	0.531	−11.72	[−21.82, −1.63]	0.023 (0.125)
PAE status × Global efficiency	22.33	[−7.30, 51.95]	0.137	15.63	[−10.82, 42.08]	0.243	22.70	[1.57, 43.83]	0.036 (0.205)
Local efficiency									
Age	0.31	[−0.02, 0.63]	0.065	0.28	[−0.05, 0.61]	0.090	0.36	[0.03–0.69]	0.035 (0.105)
PAE status	−12.71	[−29.55, 4.13]	0.137	−6.60	[−20.55, 7.34]	0.348	−6.22	[−21.31, 8.87]	0.414
Local efficiency	−1.79	[−11.30, 7.71]	0.708	3.83	[−3.21, 10.88]	0.282	−4.21	[−11.14, 2.72]	0.230
PAE status × Local efficiency	15.18	[−5.10, 35.46]	0.140	7.19	[−8.50, 22.88]	0.364	7.03	[−9.93, 24.00]	0.411
Nodal degree									
Age	0.31	[−0.02, 0.63]	0.063	0.27	[−0.06, 0.60]	0.110	0.37	[0.06, 0.69]	0.022 (0.105)
PAE status	−9.68	[−20.83, 1.47]	0.088	−6.57	[−16.95, 3.81]	0.211	−8.19	[−15.92, −0.46]	0.038 (0.260)
Nodal degree	−0.50	[−1.18, 0.18]	0.149	0.24	[−0.63, 1.12]	0.579	−0.88	[−1.64, −0.11]	0.025 (0.125)
PAE status × Nodal degree	1.66	[−0.25, 3.57]	0.087	1.23	[−0.75, 3.21]	0.220	1.70	[0.13, 3.27]	0.035 (0.205)
Betweenness centrality									
Age	0.32	[−0.01, 0.64]	0.054	0.27	[−0.06, 0.60]	0.104	0.40	[0.08, 0.72]	0.016 (0.105)
PAE status	3.87	[−1.76, 9.50]	0.175	1.54	[−1.89, 4.96]	0.373	3.24	[−0.02, 6.50]	0.052
Betweenness centrality	0.12	[−0.03, 0.27]	0.119	−0.29	[−1.06, 0.48]	0.454	0.73	[0.10, 1.37]	0.023 (0.125)
PAE status × Betweenness centrality	−0.27	[−0.66, 0.11]	0.162	−0.91	[−2.62, 0.80]	0.292	−1.44	[−2.83, −0.06]	0.041 (0.205)

*Note*. *β* = beta, CI = confidence interval, *p* = *p* value, *q* = *q* value.

**Table 3.** Associations between graph theory metrics and Receptive Communication scores

Predictors	Bilateral			Left			Right		
	*β*	CI	*p*	*β*	CI	*p*	*β*	CI	*P* (*q*)
Clustering coefficient									
Age	0.24	[−0.01, 0.48]	0.061	0.23	[−0.02, 0.48]	0.065	0.25	[0.00, 0.49]	0.050 (0.081)
PAE status	−3.92	[−14.36, 6.52]	0.457	0.29	[−8.64, 9.21]	0.949	10.12	[−7.74, 9.97]	0.802
Clustering coefficient	−0.83	[−7.74, 6.08]	0.811	20.02	[−2.97, 7.01]	0.422	−3.31	[−8.15, 1.53]	0.177
PAE status × Clustering coefficient	50.35	[−8.92, 19.63]	0.457	−0.42	[−11.30, 10.46]	0.939	−1.24	[−12.20, 9.72]	0.822
Global efficiency									
Age	0.24	[−0.01, 0.49]	0.056	0.22	[−0.03, 0.46]	0.085	0.26	[0.02, 0.50]	0.037 (0.081)
PAE status	10.68	[−12.65, 16.00]	0.816	−4.69	[−22.20, 12.82]	0.595	20.57	[−11.13, 16.26]	0.710
Global efficiency	−2.40	[−9.67, 4.86]	0.512	40.99	[−3.91, 13.89]	0.267	−6.15	[−13.69, 1.39]	0.108
PAE status × Global efficiency	−2.57	[−24.87, 19.72]	0.819	50.29	[−14.81, 25.39]	0.601	−2.84	[−18.99, 13.31]	0.727
Local efficiency									
Age	0.24	[−0.01, 0.49]	0.055	0.23	[−0.01, 0.48]	0.065	0.25	[0.00, 0.50]	0.047 (0.081)
PAE status	−6.07	[−19.16, 7.02]	0.358	−0.90	[−11.91, 10.10]	0.871	−0.43	[−11.71, 10.85]	0.940
Local efficiency	−2.24	[−9.49, 5.01]	0.540	10.82	[−3.57, 7.21]	0.503	−2.75	[−7.62, 2.11]	0.263
PAE status × Local efficiency	70.34	[−8.38, 23.05]	0.355	0.94	[−11.39, 13.28]	0.879	0.60	[−12.08, 13.28]	0.925
Nodal degree									
Age	0.24	[−0.00, 0.49]	0.053	0.22	[−0.03, 0.47]	0.083	0.25	[0.01, 0.50]	0.040 (0.081)
PAE status	10.51	[−7.11, 10.13]	0.728	−2.06	[−9.95, 5.84]	0.605	10.29	[−4.63, 7.21]	0.666
Nodal degree	−0.18	[−0.69, 0.34]	0.495	0.37	[−0.29, 1.04]	0.266	−0.44	[−1.02, 0.13]	0.127
PAE status × Nodal degree	−0.25	[−1.73, 1.22]	0.734	0.38	[−1.13, 1.89]	0.619	−0.23	[−1.44, 0.97]	0.701
Betweenness centrality									
Age	0.25	[0.01, 0.50]	0.044	0.21	[−0.03, 0.46]	0.087	0.26	[0.02, 0.51]	0.033 (0.081)
PAE status	0.46	[−3.76, 4.67]	0.829	0.61	[−1.99, 3.20]	0.642	−0.20	[−2.68, 2.28]	0.871
Betweenness centrality	0.07	[−0.04, 0.18]	0.194	−0.32	[−0.90, 0.26]	0.274	0.41	[−0.06, 0.87]	0.088
PAE status × Betweenness centrality	−0.03	[−0.32, 0.26]	0.831	−0.37	[−1.67, 0.93]	0.576	0.17	[−0.89, 1.22]	0.753

*Note*. *β* = beta, CI = confidence interval, *p* = *p* value.

### PTE Effects on Language Assessments

There were no significant main effects of PTE in any of the regression models. When controlling for PTE, nominally significant moderation effects for PAE on the Expressive Communication-graph theory metric associations remained similar to the models without PTE (Table 4).

**Table 4.** Associations between graph theory metrics and Expressive Communication scores with PTE as a covariate

Predictors	Global efficiency			Nodal degree			Betweenness centrality		
	*β*	CI	*p*	*β*	CI	*p*	*β*	CI	*p*
Age	0.36	[0.03, 0.69]	0.032	0.35	[0.02, 0.67]	0.036	0.38	[0.05, 0.70]	0.026
PTE status	0.28	[−1.40, 1.97]	0.739	0.28	[−1.40, 1.97]	0.737	0.29	[−1.40, 1.98]	0.734
PAE status	−20.06	[−38.19, −1.92]	0.031	−8.81	[−16.68, −0.94]	0.029	2.90	[−0.46, 6.25]	0.089
Graph theory metric	−12.19	[−22.50, −1.88]	0.021	−0.91	[−1.69, −0.14]	0.022	0.76	[0.11, 1.41]	0.022
PAE status × Graph theory metric	23.25	[1.93, 44.57]	0.033	1.73	[0.15, 3.31]	0.033	−1.49	[−2.89, −0.09]	0.037

*Note*. *β* = beta, CI = confidence interval, *p* = *p* value, *q* = *q* value.

### Supplementary Network Analysis

The extended network analysis in the Expressive Communication models revealed similar weak PAE moderation effects on the right hemisphere global efficiency, nodal degree, and betweenness centrality that were insignificant. The complete results are reported in the Supporting Information (Supplementary Tables 1 and 2).

## DISCUSSION

In this study, we found nominally higher clustering coefficient and local efficiency in a language network in the PAE group compared to unexposed toddlers. PAE also moderated the associations between expressive language skills and graph theory metrics such that toddlers with PAE had positive associations between language scores and structural connectivity while the opposite relationship was found in controls. To our knowledge, this is the first study examining structural network connectivity in toddlers with PAE and only the fourth study looking at the structural connectome in individuals with PAE (Ghasoub et al., 2024b; Long et al., 2020; Roos et al., 2021a). Our findings shed light on the neural correlates of early language skills in toddlers with PAE and suggest the early emergence of small differentiations in brain–language relationships. These findings also demonstrate the need for further research investigating the effects of PAE on structural brain connectivity and language development longitudinally to determine how these associations change with age as children get older and language skills develop.

Toddlers with PAE had higher local efficiency and clustering coefficient in the bilateral network compared to unexposed controls, though these differences did not survive FDR correction. Clustering coefficient measures the density of connection between nodes, while local efficiency measures how well information is transferred between neighboring regions (Bullmore & Sporns, 2009; Fornito, 2016). Thus, higher local efficiency and clustering coefficient in the network indicate higher efficient information transfer and specialized processing at the local level. Our study shows that toddlers with PAE may have denser connections and better and more mature processing between language regions at the local level, possibly indicating more specialized processing of cognitive functions. These findings align with a prior study that found no significant differences in white matter connectome measures at the global level in neonates with PAE, but higher regional connectivity (Roos et al., 2021a). However, our findings contrast studies in older children (3+ yr) and adolescents with PAE that found overall weaker structural connectivity compared to unexposed controls (Ghasoub et al., 2024b; Long et al., 2020). Our results, along with those from previous connectome studies, suggest that children with PAE may have slightly higher and/or similar connectivity and specialized processing in the structural language network during the early stages of development, and then display weaker connectivity compared to controls as they get older. This is also supported by findings from tract-based analyses that show stronger white matter connectivity (higher FA and/or lower diffusivity) in young children with PAE (Donald et al., 2024; Kar et al., 2021, 2022; Roos et al., 2021b), and weaker white matter connectivity in older children and adolescents (Ghazi Sherbaf et al., 2019; Lebel et al., 2008). Future longitudinal studies will be essential to better understanding how the structural connectome changes over time in children with PAE.

PAE had weak moderation effects on the associations between Expressive Communication scores and global efficiency, nodal degree, and betweenness centrality in the right hemisphere network. Higher global efficiency, nodal degree, and lower betweenness centrality were associated with better expressive communication skills in children with PAE, while the opposite relationship was found in unexposed children. The connectome–language relationship exhibited by the PAE children in our sample is what has been previously observed in slightly older unexposed children (3+ yr; Ghasoub et al., 2024b; Lou et al., 2021) and suggests that stronger patterns of connectivity, network integration, and decentralized communication underlie better language skills in these toddlers. Interestingly, however, the controls showed opposite relationships to those observed in older cohorts of unexposed children. These effects were in the right hemisphere, while language is typically left-lateralized. Prior work has shown more right hemisphere involvement in younger children and in individuals with reading or language difficulties (Waldie et al., 2013; Zhao et al., 2016), suggesting that more connectivity in the right hemisphere may reflect a compensatory mechanism or lack of specialization. Furthermore, these findings could also reflect, at least in part, the influence of external factors such as socioeconomic status (SES), and/or the timing and duration of exposure. All participants in this study lived with their biological parents and came from low-income families. This contrasts much prior work that has examined PAE in the context of higher-income families in North America, often in children no longer living with their biological parents. While the influence of SES on brain changes is attenuated/absent in youth with PAE, our findings may not be generalizable to higher SES populations. Our findings instead may be more relevant to children with PAE from low-income contexts and living with their biological parents, a population that has been vastly understudied. This is especially important as participants from low SES contexts are more likely to have more severe FASD diagnoses, making them one of the most impacted populations by PAE (May & Gossage, 2011). Nonetheless, future longitudinal studies along with replication in other cohorts are needed to investigate this further.

Children with and without PAE performed similarly on Expressive and Receptive Communication assessments. This contrasts with studies showing receptive and expressive language deficits in older children and adolescents with PAE (Poth et al., 2023; Proven et al., 2014; Wyper & Rasmussen, 2011). Language and communication deficits tend to worsen with age in individuals with PAE (Kippin et al., 2021; Proven et al., 2014), and thus may become apparent later. It is also possible that language deficits in individuals with PAE are influenced by other prenatal (e.g., amount and timing of exposure) and postnatal (e.g., socioeconomic) factors that may not be fully captured in these studies.

The findings of our study are limited by the small sample size, especially of children with PAE. We were also unable to test the interaction effects of prenatal alcohol and tobacco exposure due to low power, but this is important to investigate in future studies. Further research is needed to mitigate these limitations by examining the effects of PAE on structural network connectivity and language development longitudinally. The diffusion tensor model is limited by its inability to capture crossing white matter fibers (Soares et al., 2013), which could be addressed in future studies using improved diffusion modeling methods such as constrained spherical deconvolution (CSD) and neurite orientation dispersion and density imaging (NODDI) models as well as multimodal approaches. However, it is worth noting that these models have been difficult to implement in pediatric populations (especially toddlers) due to factors such as longer image acquisition time.

In summary, our study unveils novel findings into the effects of PAE on structural connectivity and language development during the toddler years, a critical neurodevelopmental period that has been understudied. We show that PAE moderates the relationships between structural brain connectivity and early language skills in toddlers, complementing studies in older children and suggesting that brain alterations are present during the toddler years. However, it is important to note that these findings did not remain significant following corrections, potentially because of the limitations discussed earlier. While we did not find group differences in language performance, the small differences in brain–language relationship between exposed and unexposed children could suggest the emergence of different trajectories from a young age that may underlie and possibly lead to later language deficits. Our findings also support the need for further longitudinal research to fully capture the differences in these associations and potentially lay the groundwork for earlier identification of reading and language deficits associated with PAE.

## ACKNOWLEDGMENTS

We sincerely thank the parents and children who participated in this study. We would also like to thank the study staff in Paarl, the study data team, and the clinical and administrative staff of the Western Cape Government Health Department at Paarl Hospital and at the clinics for their support of the study.

## FUNDING INFORMATION

Mohammad Ghasoub, Hotchkiss Brain Institute (https://dx.doi.org/10.13039/100009003). Catherine J. Wedderburn, Wellcome Trust (https://dx.doi.org/10.13039/100010269), Award ID: 203525/Z/16/Z. Heather J. Zar, South African Medical Research Council (https://dx.doi.org/10.13039/501100001322). Dan J. Stein, South African Medical Research Council (https://dx.doi.org/10.13039/501100001322). Kirsten Donald, Bill and Melinda Gates Foundation (https://dx.doi.org/10.13039/100000865), Award ID: OPP 1017641. Kirsten Donald, Brain and Behavior Research Foundation (https://dx.doi.org/10.13039/100000874), Award ID: 24467. Kristen Donald, National Research Foundation (https://dx.doi.org/10.13039/501100001321). Kirsten Donald, Newton Fund (https://dx.doi.org/10.13039/100010897), Award ID: NAF002/1001. Kirsten Donald, National Institute on Alcohol Abuse and Alcoholism (https://dx.doi.org/10.13039/100000027), Award ID: R21AA023887. Kirsten Donald, Collaborative Initiative on Fetal Alcohol Spectrum Disorders, Award ID: U24 AA014811. Catherine Lebel, Canada Excellence Research Chairs, Government of Canada (https://dx.doi.org/10.13039/501100002784). Catherine Lebel, Jacobs Foundation (https://dx.doi.org/10.13039/501100003986).

## AUTHOR CONTRIBUTIONS

**Mohammad Ghasoub**: Conceptualization: Equal; Formal analysis: Lead; Validation: Lead; Visualization: Lead; Writing – original draft: Lead; Writing – review & editing: Lead. **Chloe Scholten**: Data curation: Supporting; Validation: Supporting; Writing – review & editing: Supporting. **Bryce Geeraert**: Data curation: Supporting; Validation: Supporting; Visualization: Supporting; Writing – review & editing: Supporting. **Xiangyu Long**: Formal analysis: Supporting; Methodology: Supporting; Writing – review & editing: Supporting. **Shantanu Johsi**: Conceptualization: Supporting; Data curation: Supporting; Writing – review & editing: Supporting. **Catherine J. Wedderburn**: Conceptualization: Supporting; Writing – review & editing: Supporting. **Annerine Roos**: Data curation: Supporting; Writing – review & editing: Supporting. **Sivenesi Subramoney**: Data curation: Supporting; Writing – review & editing: Supporting. **Nadia Hoffman**: Data curation: Supporting; Writing – review & editing: Supporting. **Katherine Narr**: Conceptualization: Supporting; Writing – review & editing: Supporting. **Roger Woods**: Conceptualization: Supporting; Writing – review & editing: Supporting. **Heather J. Zar**: Conceptualization: Supporting; Writing – review & editing: Supporting. **Dan J. Stein**: Conceptualization: Supporting; Writing – review & editing: Supporting. **Kirsten Donald**: Conceptualization: Equal; Data curation: Supporting; Funding acquisition: Equal; Supervision: Supporting; Writing – review & editing: Supporting. **Catherine Lebel**: Conceptualization: Equal; Funding acquisition: Lead; Supervision: Lead; Writing – review & editing: Equal.

## DATA AND CODE AVAILABILITY STATEMENT

Requests to access the dataset in this study should be directed to kirsty.donald@uct.ac.za. Analysis code is available at: https://github.com/ghasoub-m/CT_Connectome_manuscript.

## Supplementary Material



## TECHNICAL TERMS

Teratogen: Any substance, condition, or factor that can interfere with fetal development during pregnancy and cause developmental problems (e.g., alcohol).
Diffusion weighted imaging (DWI): A brain imaging technique that measures how water moves (i.e., diffuses) in brain tissues.
Fractional anisotropy (FA): A measure of the directionality of water movement in the brain.
Mean diffusivity (MD): A measure of overall water movement in the brain.
Connectome: A detailed map of the brain regions in the brain and the connections between them.
Tractography: A technique used to reconstruct and visualize white matter pathways in the brain.

## References

[bib1] Adnams, C. M., Sorour, P., Kalberg, W. O., Kodituwakku, P., Perold, M. D., Kotze, A., September, S., Castle, B., Gossage, J., & May, P. A. (2007). Language and literacy outcomes from a pilot intervention study for children with fetal alcohol spectrum disorders in South Africa. Alcohol, 41(6), 403–414. 10.1016/j.alcohol.2007.07.005, 17936509 PMC2098695

[bib2] Almeida, L., Andreu-Fernández, V., Navarro-Tapia, E., Aras-López, R., Serra-Delgado, M., Martínez, L., García-Algar, O., & Gómez-Roig, M. D. (2020). Murine models for the study of fetal alcohol spectrum disorders: An overview. Frontiers in Pediatrics, 8, Article 359. 10.3389/fped.2020.00359, 32760684 PMC7373736

[bib3] Bathelt, J., Gathercole, S. E., Butterfield, S., the CALM team, & Astle, D. E. (2018). Children’s academic attainment is linked to the global organization of the white matter connectome. Developmental Science, 21(5), Article e12662. 10.1111/desc.12662, 29532626 PMC6175394

[bib4] Bayley, N. (2012). Bayley scales of infant and toddler development, third edition. Psychological Corporation. 10.1037/t14978-000

[bib5] Beaulieu, C. (2002). The basis of anisotropic water diffusion in the nervous system—A technical review. NMR in Biomedicine, 15(7–8), 435–455. 10.1002/nbm.782, 12489094

[bib6] Bullmore, E., & Sporns, O. (2009). Complex brain networks: Graph theoretical analysis of structural and functional systems. Nature Reviews Neuroscience, 10(3), 186–198. 10.1038/nrn2575, 19190637

[bib7] Carter, J. C., Lanham, D. C., Cutting, L. E., Clements-Stephens, A. M., Chen, X., Hadzipasic, M., Kim, J., Denckla, M. B., & Kaufmann, W. E. (2009). A dual DTI approach to analyzing white matter in children with dyslexia. Psychiatry Research: Neuroimaging, 172(3), 215–219. 10.1016/j.pscychresns.2008.09.005, 19346108 PMC2720547

[bib8] Davies, L., Dunn, M., Chersich, M., Urban, M., Chetty, C., Olivier, L., & Viljoen, D. (2011). Developmental delay of infants and young children with and without fetal alcohol spectrum disorder in the Northern Cape Province, South Africa. African Journal of Psychiatry, 14(4), 298–305. 10.4314/ajpsy.v14i4.7, 22038428

[bib9] Donald, K. A., Hendrikse, C. J., Roos, A., Wedderburn, C. J., Subramoney, S., Ringshaw, J. E., Bradford, L., Hoffman, N., Burd, T., Narr, K. L., Woods, R. P., Zar, H. J., Joshi, S. H., & Stein, D. J. (2024). Prenatal alcohol exposure and white matter microstructural changes across the first 6–7 years of life: A longitudinal diffusion tensor imaging study of a South African birth cohort. NeuroImage: Clinical, 41, Article 103572. 10.1016/j.nicl.2024.103572, 38309186 PMC10847766

[bib10] Donald, K. A., Hoogenhout, M., du Plooy, C. P., Wedderburn, C. J., Nhapi, R. T., Barnett, W., Hoffman, N., Malcolm-Smith, S., Zar, H. J., & Stein, D. J. (2018). Drakenstein Child Health Study (DCHS): Investigating determinants of early child development and cognition. BMJ Paediatrics Open, 2(1), Article e000282. 10.1136/bmjpo-2018-000282, 29942867 PMC6014194

[bib11] Fan, J., Jacobson, S. W., Taylor, P. A., Molteno, C. D., Dodge, N. C., Stanton, M. E., Jacobson, J. L., & Meintjes, E. M. (2016). White matter deficits mediate effects of prenatal alcohol exposure on cognitive development in childhood. Human Brain Mapping, 37(8), 2943–2958. 10.1002/hbm.23218, 27219850 PMC5035386

[bib12] Feng, K., Rowell, A. C., Andres, A., Bellando, B. J., Lou, X., Glasier, C. M., Ramakrishnaiah, R. H., Badger, T. M., & Ou, X. (2019). Diffusion tensor MRI of white matter of healthy full-term newborns: Relationship to neurodevelopmental outcomes. Radiology, 292(1), 179–187. 10.1148/radiol.2019182564, 31161971 PMC6614910

[bib13] Flannigan, K., Pei, J., McLachlan, K., Harding, K., Mela, M., Cook, J., Badry, D., & McFarlane, A. (2022). Responding to the unique complexities of fetal alcohol spectrum disorder. Frontiers in Psychology, 12, Article 778471. 10.3389/fpsyg.2021.778471, 35145454 PMC8821085

[bib14] Fornito, A. (2016). Graph theoretic analysis of human brain networks. In M. Filippi (Ed.), fMRI techniques and protocols (pp. 283–314). Springer. 10.1007/978-1-4939-5611-1_10

[bib15] Ghasoub, M., Perdue, M., Long, X., Donnici, C., Dewey, D., & Lebel, C. (2024a). Structural neural connectivity correlates with pre-reading abilities in preschool children. Developmental Cognitive Neuroscience, 65, Article 101332. 10.1016/j.dcn.2023.101332, 38171053 PMC10793080

[bib16] Ghasoub, M., Perdue, M., Long, X., Donnici, C., Kar, P., Gibbard, B., Tortorelli, C., McMorris, C., Dewey, D., & Lebel, C. (2024b). The brain’s structural connectivity and pre-reading abilities in young children with prenatal alcohol exposure. Developmental Cognitive Neuroscience, 70, Article 101467. 10.1016/j.dcn.2024.101467, 39486389 PMC11564048

[bib17] Ghazi Sherbaf, F., Aarabi, M. H., Hosein Yazdi, M., & Haghshomar, M. (2019). White matter microstructure in fetal alcohol spectrum disorders: A systematic review of diffusion tensor imaging studies. Human Brain Mapping, 40(3), 1017–1036. 10.1002/hbm.24409, 30289588 PMC6865781

[bib18] Girault, J. B., Cornea, E., Goldman, B. D., Knickmeyer, R. C., Styner, M., & Gilmore, J. H. (2019). White matter microstructural development and cognitive ability in the first 2 years of life. Human Brain Mapping, 40(4), 1195–1210. 10.1002/hbm.24439, 30353962 PMC6852619

[bib19] Glass, L., Graham, D. M., Akshoomoff, N., & Mattson, S. N. (2015). Cognitive factors contributing to spelling performance in children with prenatal alcohol exposure. Neuropsychology, 29(6), 817–828. 10.1037/neu0000185, 25643217 PMC4522410

[bib20] Gómez, M. J. C., Beaulieu, C., McMorris, C. A., Gibbard, B., Tortorelli, C., & Lebel, C. (2022). Frontoparietal and temporal white matter diffusion MRI in children and youth with prenatal alcohol exposure. Alcoholism: Clinical and Experimental Research, 46(10), 1808–1818. 10.1111/acer.14929, 36016474

[bib21] Hendricks, G., Malcolm-Smith, S., Adnams, C., Stein, D. J., & Donald, K. A. M. (2019). Effects of prenatal alcohol exposure on language, speech and communication outcomes: A review longitudinal studies. Acta Neuropsychiatrica, 31(2), 74–83. 10.1017/neu.2018.28, 30449293 PMC7056946

[bib22] Hendricks, G., Malcolm-Smith, S., Stein, D. J., Zar, H. J., Wedderburn, C. J., Nhapi, R. T., Chivese, T., Adnams, C. M., & Donald, K. A. (2020). Prenatal alcohol exposure is associated with early motor, but not language development in a South African cohort. Acta Neuropsychiatrica, 32(3), 145–152. 10.1017/neu.2019.51, 31902391 PMC7282868

[bib23] Hertrich, I., Dietrich, S., & Ackermann, H. (2020). The margins of the language network in the brain. Frontiers in Communication, 5, Article 519955. 10.3389/fcomm.2020.519955

[bib24] Jenkinson, M., Beckmann, C. F., Behrens, T. E. J., Woolrich, M. W., & Smith, S. M. (2012). FSL. NeuroImage, 62(2), 782–790. 10.1016/j.neuroimage.2011.09.015, 21979382

[bib25] Kar, P., Reynolds, J. E., Gibbard, W. B., McMorris, C., Tortorelli, C., & Lebel, C. (2022). Trajectories of brain white matter development in young children with prenatal alcohol exposure. Human Brain Mapping, 43(13), 4145–4157. 10.1002/hbm.25944, 35596624 PMC9374879

[bib26] Kar, P., Reynolds, J. E., Grohs, M. N., Gibbard, W. B., McMorris, C., Tortorelli, C., & Lebel, C. (2021). White matter alterations in young children with prenatal alcohol exposure. Developmental Neurobiology, 81(4), 400–410. 10.1002/dneu.22821, 33829663

[bib27] Kippin, N. R., Leitão, S., Watkins, R., & Finlay-Jones, A. (2021). Oral and written communication skills of adolescents with prenatal alcohol exposure (PAE) compared with those with no/low PAE: A systematic review. International Journal of Language & Communication Disorders, 56(4), 694–718. 10.1111/1460-6984.12644, 34137136 PMC9292204

[bib28] Lebel, C., Rasmussen, C., Wyper, K., Walker, L., Andrew, G., Yager, J., & Beaulieu, C. (2008). Brain diffusion abnormalities in children with fetal alcohol spectrum disorder. Alcoholism: Clinical and Experimental Research, 32(10), 1732–1740. 10.1111/j.1530-0277.2008.00750.x, 18671811

[bib29] Lebel, C., Roussotte, F., & Sowell, E. R. (2011). Imaging the impact of prenatal alcohol exposure on the structure of the developing human brain. Neuropsychology Review, 21(2), 102–118. 10.1007/s11065-011-9163-0, 21369875 PMC3098972

[bib30] Lebel, C., Shaywitz, B., Holahan, J., Shaywitz, S., Marchione, K., & Beaulieu, C. (2013). Diffusion tensor imaging correlates of reading ability in dysfluent and non-impaired readers. Brain and Language, 125(2), 215–222. 10.1016/j.bandl.2012.10.009, 23290366

[bib31] Leemans, A., Jeurissen, B., Sijbers, J., & Jones, D. K. (2009). ExploreDTI: A graphical toolbox for processing, analyzing, and visualizing diffusion MR data [Abstract]. In ISMRM 17th Scientific Meeting and Exhibition. MIRA. https://cds.ismrm.org/protected/09MProceedings/PDFfiles/03537.pdf

[bib32] Lindinger, N. M., Jacobson, S. W., Davidson, L., Conradie, S., Dodge, N. C., Molteno, C. D., Meintjes, E. M., Gaab, N., & Jacobson, J. L. (2022). Reading impairment in adolescents with fetal alcohol spectrum disorders. Scientific Studies of Reading, 26(6), 469–488. 10.1080/10888438.2022.2054717, 36388467 PMC9642985

[bib33] Long, X., Little, G., Treit, S., Beaulieu, C., Gong, G., & Lebel, C. (2020). Altered brain white matter connectome in children and adolescents with prenatal alcohol exposure. Brain Structure and Function, 225(3), 1123–1133. 10.1007/s00429-020-02064-z, 32239277

[bib34] Lou, C., Cross, A. M., Peters, L., Ansari, D., & Joanisse, M. F. (2021). Rich-club structure contributes to individual variance of reading skills via feeder connections in children with reading disabilities. Developmental Cognitive Neuroscience, 49, Article 100957. 10.1016/j.dcn.2021.100957, 33894677 PMC8093404

[bib35] Mao, J., Liu, L., Perkins, K., & Cao, F. (2021). Poor reading is characterized by a more connected network with wrong hubs. Brain and Language, 220, Article 104983. 10.1016/j.bandl.2021.104983, 34174464

[bib36] Martin, A., Schurz, M., Kronbichler, M., & Richlan, F. (2015). Reading in the brain of children and adults: A meta-analysis of 40 functional magnetic resonance imaging studies. Human Brain Mapping, 36(5), 1963–1981. 10.1002/hbm.22749, 25628041 PMC4950303

[bib37] May, P. A., & Gossage, J. P. (2011). Maternal risk factors for fetal alcohol spectrum disorders: Not as simple as it might seem. Alcohol Research & Health, 34(1), 15–26. 23580036 PMC3860552

[bib38] McLachlan, K., Zhou, D., Little, G., Rasmussen, C., Pei, J., Andrew, G., Reynolds, J. N., & Beaulieu, C. (2020). Current socioeconomic status correlates with brain volumes in healthy children and adolescents but not in children with prenatal alcohol exposure. Frontiers in Human Neuroscience, 14, Article 223. 10.3389/fnhum.2020.00223, 32714166 PMC7344164

[bib39] Moore, E. M., & Xia, Y. (2022). Neurodevelopmental trajectories following prenatal alcohol exposure. Frontiers in Human Neuroscience, 15, Article 695855. 10.3389/fnhum.2021.695855, 35058760 PMC8763806

[bib40] Nikki Arrington, C., Kulesz, P. A., Juranek, J., Cirino, P. T., & Fletcher, J. M. (2017). White matter microstructure integrity in relation to reading proficiency. Brain and Language, 174, 103–111. 10.1016/j.bandl.2017.08.002, 28818624 PMC5617339

[bib41] Niogi, S. N., & McCandliss, B. D. (2006). Left lateralized white matter microstructure accounts for individual differences in reading ability and disability. Neuropsychologia, 44(11), 2178–2188. 10.1016/j.neuropsychologia.2006.01.011, 16524602

[bib42] Popova, S., Charness, M. E., Burd, L., Crawford, A., Hoyme, H. E., Mukherjee, R. A. S., Riley, E. P., & Elliott, E. J. (2023). Fetal alcohol spectrum disorders. Nature Reviews Disease Primers, 9(1), Article 11. 10.1038/s41572-023-00420-x, 36823161

[bib43] Popova, S., Lange, S., Shield, K., Mihic, A., Chudley, A. E., Mukherjee, R. A. S., Bekmuradov, D., & Rehm, J. (2016). Comorbidity of fetal alcohol spectrum disorder: A systematic review and meta-analysis. Lancet, 387(10022), 978–987. 10.1016/S0140-6736(15)01345-8, 26777270

[bib44] Poth, L. D., Love, T., & Mattson, S. N. (2023). Profiles of language and communication abilities in adolescents with fetal alcohol spectrum disorders. Journal of the International Neuropsychological Society, 29(8), 724–733. 10.1017/S1355617722000789, 36325639 PMC10154428

[bib45] Proven, S., Ens, C., & Beaudin, P. G. (2014). The language profile of school-aged children with fetal alcohol spectrum disorder (FASD). Canadian Journal of Speech-Language Pathology & Audiology, 37(4), 268–279.

[bib46] R Core Team. (2021). R: A language and environment for statistical computing (Version 4.2.1) [Software]. R Foundation for Statistical Computing. https://www.R-project.org/

[bib47] Rademeyer, V., & Jacklin, L. (2013). A study to evaluate the performance of black South African urban infants on the Bayley Scales of Infant Development III. South African Journal of Child Health, 7(2), 54–59. 10.7196/sajch.547

[bib48] Roos, A., Fouche, J.-P., Ipser, J. C., Narr, K. L., Woods, R. P., Zar, H. J., Stein, D. J., & Donald, K. A. (2021a). Structural and functional brain network alterations in prenatal alcohol exposed neonates. Brain Imaging and Behavior, 15(2), 689–699. 10.1007/s11682-020-00277-8, 32306280 PMC7572489

[bib49] Roos, A., Wedderburn, C. J., Fouche, J.-P., Subramoney, S., Joshi, S. H., Woods, R. P., Zar, H. J., Narr, K. L., Stein, D. J., & Donald, K. A. (2021b). Central white matter integrity alterations in 2–3-year-old children following prenatal alcohol exposure. Drug and Alcohol Dependence, 225, Article 108826. 10.1016/j.drugalcdep.2021.108826, 34182371 PMC8299546

[bib50] Rubinov, M., & Sporns, O. (2010). Complex network measures of brain connectivity: Uses and interpretations. NeuroImage, 52(3), 1059–1069. 10.1016/j.neuroimage.2009.10.003, 19819337

[bib51] Sket, G. M., Overfeld, J., Styner, M., Gilmore, J. H., Entringer, S., Wadhwa, P. D., Rasmussen, J. M., & Buss, C. (2019). Neonatal white matter maturation is associated with infant language development. Frontiers in Human Neuroscience, 13, Article 434. 10.3389/fnhum.2019.00434, 31920593 PMC6927985

[bib52] Soares, J., Marques, P., Alves, V., & Sousa, N. (2013). A hitchhiker’s guide to diffusion tensor imaging. Frontiers in Neuroscience, 7, Article 31. 10.3389/fnins.2013.00031, 23486659 PMC3594764

[bib53] Sowell, E. R., Johnson, A., Kan, E., Lu, L. H., Van Horn, J. D., Toga, A. W., O’Connor, M. J., & Bookheimer, S. Y. (2008). Mapping white matter integrity and neurobehavioral correlates in children with fetal alcohol spectrum disorders. Journal of Neuroscience, 28(6), 1313–1319. 10.1523/JNEUROSCI.5067-07.2008, 18256251 PMC3567846

[bib54] Stein, D. J., Koen, N., Donald, K. A., Adnams, C. M., Koopowitz, S., Lund, C., Marais, A., Myers, B., Roos, A., Sorsdahl, K., Stern, M., Tomlinson, M., van der Westhuizen, C., Vythilingum, B., Myer, L., Barnett, W., Britten, K., & Zar, H. J. (2015). Investigating the psychosocial determinants of child health in Africa: The Drakenstein Child Health Study. Journal of Neuroscience Methods, 252, 27–35. 10.1016/j.jneumeth.2015.03.016, 25797842 PMC4556362

[bib55] Travis, K. E., Adams, J. N., Kovachy, V. N., Ben-Shachar, M., & Feldman, H. M. (2017). White matter properties differ in 6-year old readers and pre-readers. Brain Structure and Function, 222(4), 1685–1703. 10.1007/s00429-016-1302-1, 27631434 PMC5352545

[bib56] Treit, S., Lebel, C., Baugh, L., Rasmussen, C., Andrew, G., & Beaulieu, C. (2013). Longitudinal MRI reveals altered trajectory of brain development during childhood and adolescence in fetal alcohol spectrum disorders. Journal of Neuroscience, 33(24), 10098–10109. 10.1523/JNEUROSCI.5004-12.2013, 23761905 PMC6618394

[bib57] Turker, S., Reiterer, S. M., Schneider, P., & Seither-Preisler, A. (2019). Auditory cortex morphology predicts language learning potential in children and teenagers. Frontiers in Neuroscience, 13, Article 824. 10.3389/fnins.2019.00824, 31447639 PMC6692463

[bib58] Tzourio-Mazoyer, N., Landeau, B., Papathanassiou, D., Crivello, F., Etard, O., Delcroix, N., Mazoyer, B., & Joliot, M. (2002). Automated anatomical labeling of activations in SPM using a macroscopic anatomical parcellation of the MNI MRI single-subject brain. NeuroImage, 15(1), 273–289. 10.1006/nimg.2001.0978, 11771995

[bib59] Vourkas, M., Micheloyannis, S., Simos, P. G., Rezaie, R., Fletcher, J. M., Cirino, P. T., & Papanicolaou, A. C. (2011). Dynamic task-specific brain network connectivity in children with severe reading difficulties. Neuroscience Letters, 488(2), 123–128. 10.1016/j.neulet.2010.11.013, 21073917 PMC3014432

[bib60] Waldie, K. E., Haigh, C. E., Badzakova-Trajkov, G., Buckley, J., & Kirk, I. J. (2013). Reading the wrong way with the right hemisphere. Brain Sciences, 3(3), 1060–1075. 10.3390/brainsci3031060, 24961521 PMC4061874

[bib61] Wang, N. Y.-H., Wang, H.-L. S., Liu, Y.-C., Chang, Y.-P. E., & Weng, J.-C. (2021). Investigating the white matter correlates of reading performance: Evidence from Chinese children with reading difficulties. PLOS ONE, 16(3), Article e0248434. 10.1371/journal.pone.0248434, 33705494 PMC7951916

[bib62] Wyper, K. R., & Rasmussen, C. R. (2011). Language impairments in children with fetal alcohol spectrum disorder. Journal of Population Therapeutics and Clinical Pharmacology, 18(2), e364–e376. https://www.jptcp.com/index.php/jptcp/article/view/485/413, 21712561

[bib63] Xia, M., Wang, J., & He, Y. (2013). BrainNet Viewer: A network visualization tool for human brain connectomics. PLOS ONE, 8(7), Article e68910. 10.1371/journal.pone.0068910, 23861951 PMC3701683

[bib64] Yu, X., Dunstan, J., Jacobson, S. W., Molteno, C. D., Lindinger, N. M., Turesky, T. K., Meintjes, E. M., Jacobson, J. L., & Gaab, N. (2022). Distinctive neural correlates of phonological and reading impairment in fetal alcohol-exposed adolescents with and without facial dysmorphology. Neuropsychologia, 169, Article 108188. 10.1016/j.neuropsychologia.2022.108188, 35218791 PMC9922095

[bib65] Zhao, J., Thiebaut de Schotten, M., Altarelli, I., Dubois, J., & Ramus, F. (2016). Altered hemispheric lateralization of white matter pathways in developmental dyslexia: Evidence from spherical deconvolution tractography. Cortex, 76, 51–62. 10.1016/j.cortex.2015.12.004, 26859852

