# Supplementary material for: The Effects of Prenatal Alcohol Exposure on Structural Brain Connectivity and Early Language Skills in a South African Birth Cohort
Source: Neurobiol Lang (Camb). 2025 Apr 2;6:nol_a_00161. doi: 10.1162/nol_a_00161 (PMC11977823; doi:10.1162/nol_a_00161)
Supplement: Supplementary file 1 [file nol-6-1-161-s001.pdf]

Supplementary Table 1: Associations between graph theory metrics and Expressive Communication scores (β = Beta, p = p-value)

	**Bilateral**		**Left**		**Right**	
Predictors	β	*p*	β	*p*	β	*p*
(Intercept)	-6.69	0.368	-8.08	0.248	-2.74	0.704
sex	0.31	0.575	0.31	0.584	0.22	0.693
Age	0.31	0.065	0.30	0.070	0.33	0.053
household income	0.77	0.081	0.87	**0.048**	0.88	**0.049**
PAE	-8.90	0.318	-2.48	0.733	-5.39	0.589
Clustering Coefficient	3.81	0.553	5.22	0.337	-2.22	0.694
Clustering Coefficient × PAE	11.46	0.328	2.99	0.734	6.38	0.593

	**Bilateral**		**Left**		**Right**	
Predictors	β	*p*	β	*p*	β	*p*
(Intercept)	0.37	0.958	-7.99	0.362	5.48	0.466
sex	0.27	0.630	0.42	0.445	0.26	0.629
Age	0.31	0.065	0.26	0.111	0.37	**0.026**
household income	0.83	0.061	0.87	**0.044**	0.74	0.088
PAE	-11.84	0.259	-16.79	0.176	-21.78	0.056
Global Efficiency	-6.75	0.307	6.32	0.416	-13.34	0.063
Global Efficiency × PAE	18.54	0.262	19.20	0.179	25.47	0.056

	**Bilateral**		**Left**		**Right**	
Predictors	β	*p*	β	*p*	β	*p*

(Intercept)	-4.88	0.581	-7.89	0.314	-3.52	0.661
sex	0.40	0.480	0.35	0.527	0.23	0.680
Age	0.31	0.065	0.30	0.068	0.33	**0.049**
household income	0.72	0.103	0.80	0.069	0.83	0.060
PAE	-16.12	0.160	-6.65	0.470	-16.19	0.237
Local Efficiency	1.21	0.871	4.57	0.470	-1.24	0.842
Local Efficiency × PAE	18.58	0.164	7.37	0.471	17.64	0.240

	**Bilateral**		**Left**		**Right**	
Predictors	β	*p*	β	*p*	β	*p*
(Intercept)	-1.21	0.842	-4.97	0.426	-1.02	0.858
sex	0.31	0.577	0.41	0.459	0.25	0.645
Age	0.30	0.071	0.26	0.112	0.36	**0.028**
household income	0.79	0.075	0.88	**0.042**	0.76	0.082
PAE	-9.32	0.149	-7.20	0.188	-8.96	0.071
Nodal Degree	-0.34	0.323	0.37	0.402	-0.75	0.069
Nodal Degree × PAE	1.28	0.150	1.06	0.192	1.40	0.070

	**Bilateral**		**Left**		**Right**	
Predictors	β	*p*	β	*p*	β	*p*
(Intercept)	-5.18	0.356	-1.81	0.739	-7.90	0.162
sex	0.25	0.649	0.44	0.423	0.28	0.601
Age	0.31	0.064	0.26	0.108	0.37	**0.024**
household income	0.87	**0.049**	0.85	**0.049**	0.71	0.101
PAE	2.96	0.332	2.41	0.198	3.69	0.052

Betweenness Centrality	0.06	0.343	-0.31	0.445	0.70	0.057
Betweenness Centrality × PAE	-0.16	0.309	-1.05	0.158	-1.40	**0.040**

Supplementary Table 2: Associations between graph theory metrics and Receptive Communication scores (β = Beta, p = p-value)

	**Bilateral**		**Left**		**Right**	
Predictors	β	*p*	β	*p*	β	*p*
(Intercept)	-1.59	0.773	-4.09	0.443	0.67	0.900
sex	-0.17	0.689	-0.19	0.648	-0.21	0.619
Age	0.22	0.074	0.23	0.070	0.24	0.057
household income	0.35	0.285	0.42	0.205	0.42	0.198
PAE	-7.05	0.297	-0.73	0.897	-1.68	0.819
Clustering Coefficient	1.10	0.815	3.82	0.352	-2.47	0.561
Clustering Coefficient × PAE	9.23	0.299	0.94	0.890	2.05	0.817

	**Bilateral**		**Left**		**Right**	
Predictors	*B*	*p*	*B*	*p*	*B*	*p*
(Intercept)	-0.06	0.990	-5.84	0.383	5.21	0.364
sex	-0.24	0.566	-0.11	0.793	-0.25	0.541
Age	0.24	0.062	0.20	0.100	0.26	**0.034**
household income	0.43	0.190	0.42	0.186	0.38	0.236
PAE	0.17	0.982	-9.05	0.338	-1.49	0.862
Global Efficiency	-1.87	0.652	6.51	0.271	-8.62	0.108

Global Efficiency × PAE	-0.28	0.982	10.40	0.338	1.84	0.854

	**Bilateral**		**Left**		**Right**	
Predictors	β	*p*	β	*p*	β	*p*
(Intercept)	0.17	0.980	-4.39	0.463	0.00	1.000
sex	-0.09	0.823	-0.16	0.697	-0.23	0.588
Age	0.22	0.072	0.23	0.069	0.24	0.055
household income	0.32	0.328	0.37	0.257	0.40	0.224
PAE	-12.16	0.162	-2.94	0.678	-7.88	0.443
Local Efficiency	-1.17	0.833	3.86	0.421	-1.52	0.744
Local Efficiency × PAE	14.12	0.162	3.31	0.673	8.65	0.443

	**Bilateral**		**Left**		**Right**	
Predictors	β	*p*	β	*p*	β	*p*
(Intercept)	-0.25	0.957	-2.67	0.574	0.97	0.821
sex	-0.24	0.569	-0.12	0.775	-0.26	0.531
Age	0.24	0.059	0.20	0.101	0.26	**0.034**
household income	0.40	0.219	0.43	0.179	0.39	0.228
PAE	0.04	0.993	-3.85	0.355	-0.14	0.971
Nodal Degree	-0.14	0.575	0.37	0.270	-0.49	0.115
Nodal Degree × PAE	-0.01	0.992	0.58	0.354	0.03	0.952

	**Bilateral**		**Left**		**Right**	
Predictors	β	*p*	β	*p*	β	*p*
(Intercept)	-2.29	0.587	0.60	0.884	-3.39	0.425
sex	-0.22	0.602	-0.09	0.823	-0.24	0.560

Age	0.24	0.054	0.20	0.098	0.26	**0.034**
household income	0.38	0.248	0.41	0.200	0.37	0.250
PAE	0.81	0.713	1.36	0.340	0.61	0.673
Betweenness Centrality	0.05	0.294	-0.34	0.275	0.45	0.099
Betweenness Centrality × PAE	-0.04	0.698	-0.57	0.312	-0.20	0.694
